# Detection of Antagonistic Compounds Synthesized by *Bacillus velezensis* against *Xanthomonas citri* subsp. *citri* by Metabolome and RNA Sequencing

**DOI:** 10.3390/microorganisms11061523

**Published:** 2023-06-08

**Authors:** Muhammad Fazle Rabbee, Kwang-Hyun Baek

**Affiliations:** Department of Biotechnology, Yeungnam University, Gyeongsan 38541, Republic of Korea; rabbi.biotech@gmail.com

**Keywords:** *B. velezensis*, *Xanthomonas citri* subsp. *citri*, lipopeptides, polyketides, gene expression, bacilysin

## Abstract

Biological control of plant diseases has gained attraction for controlling various bacterial diseases at a field trial stage. An isolated endophytic bacterium, *Bacillus velezensis* 25 (Bv-25), from *Citrus* species had strong antagonistic activity against *Xanthomonas citri* subsp. *citri* (*Xcc*), which causes citrus canker disease. When Bv-25 was incubated in Landy broth or yeast nutrient broth (YNB), the ethyl acetate extract of Landy broth exhibited higher levels of antagonistic activity against *Xcc* compared to that of YNB. Therefore, the antimicrobial compounds in the two ethyl acetate extracts were detected by high performance liquid chromatography–mass spectrometry. This comparison revealed an increase in production of several antimicrobial compounds, including difficidin, surfactin, fengycin, and Iturin-A or bacillomycin-D by incubation in Landy broth. RNA sequencing for the Bv-25 grown in Landy broth were performed, and the differential expressions were detected for the genes encoding the enzymes for the synthesis of antimicrobial compounds, such as bacilysin, plipastatin or fengycin, surfactin, and mycosubtilin. Combination of metabolomics analysis and RNA sequencing strongly suggests that several antagonistic compounds, especially bacilysin produced by *B. velezensis*, exhibit an antagonistic effect against *Xcc*.

## 1. Introduction

Phytopathogenic bacteria cause numerous plant diseases worldwide, resulting in considerable losses in crop production and storage [1]. The control strategies for these diseases pose one of the greatest challenges to sustainable agriculture. Due to the difficulty of managing bacterial diseases naturally, growers heavily rely on chemical agents, especially pesticides [2]. Extensive and repeated applications of these chemicals have led to the development of pesticide resistance in the target phytopathogens and even non-target organisms, including human beings [2]. Besides chemical pesticides, the public is becoming more concerned about using antibiotics in the environment due to their adverse effects on human health and are suspicious of the massive application of antibiotics due to the advent of antibacterial resistance [3]. Therefore, in recent years, biological control agents (i.e., specialized microbes or its metabolite to control plant diseases) are gaining attention from scientists and farmers as an alternative mean of plant disease management. Until now, numerous biocontrol agents have been commercially available to control plant diseases [4].

Citrus canker is a devastating disease caused by the bacterial pathogen *Xanthomonas citri* subsp. *citri* (*Xcc*), which affects most commercial citrus varieties. Infection of *Xcc* causes necrotic lesions on citrus leaves, stems, and fruits, resulting in premature fruit loss, defoliation, twig dieback, and tree decline [5]. The disease impacts fruit production detrimentally by both decreasing fruit yields in most citrus-producing countries and making the infected fruits less valuable and unmarketable [6]. The most common approach to control citrus canker is the usage of copper-based bactericidal sprays such as copper hydroxide, cuprous oxide, and copper oxychloride [7]. Copper-resistant strains, however, have been detected on several phytopathogenic bacteria, including *Xcc*, *X. axonopodis* pv. *vesicatoria*, *X. alfalfae* subsp. *citrumelonis*, and *Pseudomonas syringae* pv. *tomato* [7,8]. A copper-resistant strain of *Xcc* was first reported in 1994 in the province of Corrientes, Argentina, where the citrus nurseries were exposed to regular application of copper-based bactericides [8]. The antibiotic streptomycin is extensively used to control *Xcc*; however, streptomycin resistance emerges several- or thousand-folds due to chromosomal or gene mutations [9]. Streptomycin resistance exhibits an increasing pattern due to the repeated usage of the chemical. For example, in the case of pathogenic *X. perforans*, resistance increased from 5% in 2006 to 14% in 2011 during field applications of streptomycin [10]. 

Plant pathologists and microbiologists, therefore, have developed novel strategies for developing biocontrol agents to control phytopathogenic bacteria in more cost-effective and environmentally friendly ways [11]. Numerous new microbes containing biologically active agents have been identified in an effort to reduce the excessive use of chemical pesticides in agriculture [12]. Among the commercially developed biocontrol agents, the most used organisms are based on the isolation of *Bacillus* spp. For example, Botrybel (*B. velezensis*), RhizoVital^®^ (*B. velezensis* FZB42^T^), Serenade (*B. velezensis* QST713), Kodiak ^TM^ (*B. velezensis* GB03), and Taegro^®^ (*B. velezensis* FZB24) [13]. *Pseudomonas*- and *Strepotomyces-*based biocontrol agents are also available in the market [12]. These biocontrol agents exhibit various modes of action, such as antimicrobial compound secretion, systemic resistance induction, and nutrient competition with the pathogenic microbes [11]. 

Next-generation genome sequencing has revealed that many biosynthetic gene clusters encode bioactive secondary metabolites in the genomes of several *Bacillus* species, e.g., *B. velezensis*, *B. subtilis*, *B. amyloliquefaciens*, and *B. siamensis* [14,15,16,17]. *B. velezensis* is an endospore-forming and free-living soil bacterium, first described by Ruiz-Garcia in 2005 [18]. The enzymes encoded by these biosynthetic genes synthesize antimicrobial compounds that could serve as a reservoir of novel scaffolds for drug screening to treat numerous plant diseases [19,20]. Following complete sequencing and annotation of the *B. velezensis* genome, considerable parts of the genomic DNA were found harboring various antimicrobial gene clusters. The clusters of *srf*, *bmy*, *fen*, *mln*, *dfn*, *bae*, *dhb*, and *bac* encode the active compounds surfactins, bacillomycin D, fengycins, macrolactin, difficidin, bacillaene, bacillibactin, and bacilysin, respectively [21]. These bioactive secondary metabolites have numerous applications in plant-microbe interactions with antimicrobial and nematocidal activities [21]. Among the compounds, surfactins, bacillomycin D, and fengycins are grouped as lipopeptides (LPs), and the classification of these compounds is based on the length variation of fatty acid chain and amino acid sequences of these compounds [22]. Macrolactin, difficidin, and bacillaene are classified as polyketide molecules (PKs) that are synthesized by giant modular polyketide synthases with multifunctional domains [23].

LPs are mainly associated with antifungal action, especially against filamentous fungi. For example, bacillomycin D synthesized by *B. velezensis* FZB42 exhibited strong antifungal action against *Fusarium graminearum* that causes *Fusarium* head blight [24]. Lipopeptide fengycins produced by *B. velezensis* Bs006 suppressed mycelial growth of *F. oxysporum* f. sp. *physali* that is responsible for golden berry wilt disease [25]. Surfactin B and surfactin C produced by *B. velezensis* 9D-6 exhibits antifungal activities against *Monilinia fructicola* that causes brown rot of nectarine and some other stone fruits [26]. Similarly, the antifungal activity of *B. velezensis* LM2303 was mainly associated with fengycin B, iturin A, and surfactin A [25]. Under field conditions, LM2303 exhibited strong antagonist activity against *F. graminearum* by significantly reducing disease severity of *Fusarium* head blight, with a control efficiency of 72.3% [27]. 

The antibacterial activity of *B. velezensis* is mainly associated with PKs including macrolactin, difficidin, and bacillaene [28]. For example, difficidin and bacilysin produced by *B. velezensis* FZB42 exhibited antibacterial activity against *Xanthomonas oryzae* pv. *oryzae* and *X. oryzae* pv. *oryzicola* [29]. *B. velezensis* LM2303 encodes several antibacterial compounds such as surfactin A, butirosin, plantazolicin, kijanimicin, bacilysin, difficidin, bacillaene, and macrolactin. In addition, other metabolites including iron–siderophore bacillibactin, molybdenum cofactor, and teichuronic acid were involved with nutrient uptake during plant–microbe interaction [27]. Moreover, the antifungal action of dipeptide antibiotic bacilysin was reported; for example, bacilysin produced by FZB42 damaged the hyphal structure of *Phytophthora sojae* that causes root rot disease in soybean [30]. Two other ribosomally synthesized bacteriocin, known as plantazolicin and amylocyclicin in *B. velezensis*, displayed high antibacterial activities against closely related Gram-positive bacteria [31,32]. Bacillibactin secreted by *B. velezensis* can bind with environmental iron to form siderophore–iron complex and can be internalized into the bacterium, where iron is released for cellular processes or nutritional requirements [33].

Previously, we isolated *B. velezensis*-25 (Bv-25), which exhibited antibacterial activity against wild-type and streptomycin-resistant *Xcc* strains [34,35]. Therefore, we conducted this research to further understand the antibacterial compounds produced by *B. velezensis* that can control citrus canker disease effectively and efficiently. Through the application of various state-of-the-art techniques, such as transcriptome analysis, high performance liquid chromatography–mass spectrometry (HPLC-MS) analysis, and antimicrobial assays, we identified the differentially expressed genes and antibacterial compounds, which may be involved in exerting the antagonistic effects on *Xcc*. 

## 2. Materials and Methods

### 2.1. Strains and Culture Conditions

Bv-25 and *B. amyloliquefaciens* strain 2 (Ba-2) were incubated in yeast nutrient broth (YNB: yeast extract, 5 g; and nutrient broth, 8 g in 1 L distilled water) and in Landy broth (L-glutamic acid 5 g, glucose 20 g, yeast extract 1 g, phenylalanine 2 mg, MgSO_4_·7H_2_O 0.5 g, KCl 0.5 g, MnSO_4_ 5 mg, CuSO_4_·5H_2_O 0.16 mg, FeSO_4_·7H_2_O 0.15 mg, KH_2_PO_4_ 1 g in 1 L H_2_O, pH 7.0). Bv-25 was isolated from citrus species in our laboratory, and Ba-2 was provided by the Korean Agricultural Culture Collection (KACC, RDA, Wanju-gun, Republic of Korea). The pathogenic *Xcc* strains used for bioassay were composed of two *Xcc* strains, wild-type *Xcc*W1 and streptomycin-resistant mutant *Xcc*M4 [9]. The pathogenic *Xcc* strains were kind gifts from Dr. Hyun (Citrus Research Station, NIHH, RDA, Jeju, Republic of Korea). The wild-type and streptomycin-resistant strains of *Xcc* were routinely grown on yeast nutrient agar (YNA, yeast extract, 5 g; nutrient broth, 8 g; and agar, 1.5 g in distilled water, 1 L) or YNA supplemented with 50 ppm streptomycin, respectively. All the strains, such as Bv-25, Ba-2, and *Xcc*, were maintained in YNB containing 50% glycerol at −80 °C until use. 

### 2.2. Antibacterial Activity of Ethyl Acetate Extracts of Bv-25 and Ba-2 in Landy Broth and YNB

To compare the production of active compounds in different media, 50 μL of *B. velezensis* Bv-25 and Ba-2 cells from the stock were added to 2 mL of different media including Landy broth and YNB. The cultures were then incubated for 24 h at 28 °C. The seed cultures (0.1 mL) of *B. velezensis* Bv-25 and Ba-2 were then transferred into 100 mL of Landy and YNB broth and cultured for 48 h at 28 °C in a shaking incubator with 180 rpm. The growth of bacterial cells in different media was monitored using OD600_nm_ by a spectrophotometer (ASP-3700, ACTGene, Piscataway, NJ, USA). An equal volume of ethyl acetate (100 mL) was added to the bacterial cultures, sonicated for 5 min, and maintained overnight with vigorous shaking. The culture mixture was centrifuged at 3000 rpm for 10 min at 4 °C, the supernatants collected, and dried in a rotary evaporator (A-1000S; Eyela, Tokyo, Japan) at 50 °C. 

The residues were dissolved In 1.5 mL HPLC-grade methanol and air-dried under a chemical hood. After drying, metabolites were weighed and the metabolite concentrations were prepared (10.0 mg mL^−1^, 1.0 mg mL^−1^ and 0.1 mg mL^−1^) using HPLC-grade methanol. 

Disk-diffusion assays were used to test the antibacterial activity of ethyl acetate extracts against *Xcc* strains. *Xcc*W1 and *Xcc*M4 strain grown overnight was mixed with 5 mL of 0.7% YNA soft agar and directly poured onto 1.5% agar YNA plates. After gel solidification, 30 µL of the metabolites in three different concentrations, such as 10.0 mg mL^−1^, 1.0 mg mL^−1^, and 0.1 mg mL^−1^, were placed on a sterile paper disk. The antibacterial activity of the ethyl acetate extract was compared with the positive control composed of two streptomycin concentrations (S1: streptomycin 1.0 mg mL^−1^; S2: streptomycin 0.1 mg mL^−1^) and a negative control composed of 100% methanol. The inhibition zones after 24 h incubation at 28 °C around the paper disks were measured using an electronic digital caliper, and the antibacterial activities were compared. The experiment was conducted twice with three replicates. 

### 2.3. Minimum Inhibitory Concentration (MIC) and Minimum Bactericidal Concentration (MBC) of Bv-25 and Ba-2 Extracts Cultured in Landy Broth and YNB 

After selecting Landy medium as the highest antibacterial activity-inducing medium, MIC and MBC of ethyl acetate extracts of Bv-25, Ba-2, and streptomycin were determined using Landy broth and YNB by the broth microdilution method [36]. The metabolite was collected from the bacterial culture, weighed and dissolved in methanol at a concentration of 2.0 gm mL^−1^ (stock solution). For MIC analysis, the metabolites of Bv-25 and Ba-2 were two-fold serially diluted using methanol in order to make concentrations ranging from 15.625–1000 μg mL^−1^. The study of MIC was conducted in 96-well plates. Each well contained a total 200 µL, composed of 100 µL metabolites of Bv-25 and Ba-2, 90 µL broth, and 10 µL indicator bacterial inoculum with an optical density of 2.0 at 600 nm. One well served as a positive control (YNB plus inoculum), and one served as a negative control (only YNB). The plates were incubated at 28 °C for 24 h. MIC was defined as the lowest concentration of metabolite extract that prevented the visible growth of the indicator bacterial strain. Additionally, MBC was determined by plating 10 µL of the cultures from each well in YNA agar plates. MBC was defined as the lowest concentration of metabolite extract that did not show any bacterial growth in YNA plates after an incubation period of 24 h at 28 °C. Both the MIC and MBC were expressed in µg mL^−1^. 

### 2.4. HPLC-MS Analysis of Ethyl Acetate Extracts from Bv-25 and Ba-2

The ethyl acetate extracts of *Bacillus* spp. were filtered by using 0.22 µm nylon filter that was then analyzed by using liquid chromatograph–mass spectrometry (HPLC-MS). The HPLC-MS system was composed of an HPLC apparatus (model 2695; Waters, Milford, MA, USA) equipped with a pentafluorophenyl column (Luna C18 reversed phase column, 4.6 × 150 mm, 5μm; Phenomenex, Torrance, CA, USA) and a mass spectrometer (Waters model 3100, Milford, MA, USA). The HPLC conditions were as follows: injection volume = 5 µL, solvent A = 20 mM Ammonium acetate buffer (pH adjusted to 7.2), solvent B = acetonitrile, and solvent program = 50: 50 (A/B) to 50: 50 (A/B) over 10 min at a flow rate of 0.5 mL/min. MS data was obtained under the following conditions: desolvation gas (N_2_) flow rate = 4 L/h, desolvation temperature = 350 °C, capillary voltage = 4 kV, cone voltage = 30 V, ionization mode = electrospray positive, and single ion recording *m*/*z* = 163.

### 2.5. Differential Gene Expression Analysis of Bv-25 Grown in Landy Broth and YNB

RNA-sequence analysis was employed to disclose the transcriptomic features of Bv-25 genes that were expressed in two different media (Landy and YNB). In this process, genetic information from a gene is translated in the synthesis of a functional gene product, typically proteins. Total RNA was purified from Bv-25 and propagated for 24 h using Landy broth and YNB. Bacteria were collected by centrifugation at 8000 rpm at 4 °C for 10 min, and the cell pellets (bacterial cells) were then immediately frozen in liquid nitrogen and stored at −80 °C until use. RNA extraction (performed with the RNeasy Midi kit; Qiagen, Hilden, Germany), RNA quantification, and cDNA synthesis and labeling were performed as described previously [37]. Log2 fold change (log2 FC) was calculated through dividing FPKM (Fragments per kilobase of exon model per million mapped reads) value of Bv-25 grown in Landy by Bv-25 grown on YNB to obtain fold change (FC) and then putting the equation in excel as Log(FC, 2).

### 2.6. Antibacterial Activity Assay of Pure Surfactin and Fengycin against Xcc

Several antibacterial compounds were detected by metabolome and transcriptome analysis. Several compounds such as fengycin and surfactin (purchased from Sigma-Aldrich, St. Louis, MO, USA) and macrolactin isolated from Bv-25 (purified and identified by Dr. Sanghee Shim from Seoul National University, Seoul, Republic of Korea) were tested for antagonistic effects against *Xcc* based on the procedure described in Section 2.2.

## 3. Results

### 3.1. Antibacterial Activity of Ethyl Acetate Extracts of Bv-25 in Landy and YNB

**Figure 1:**
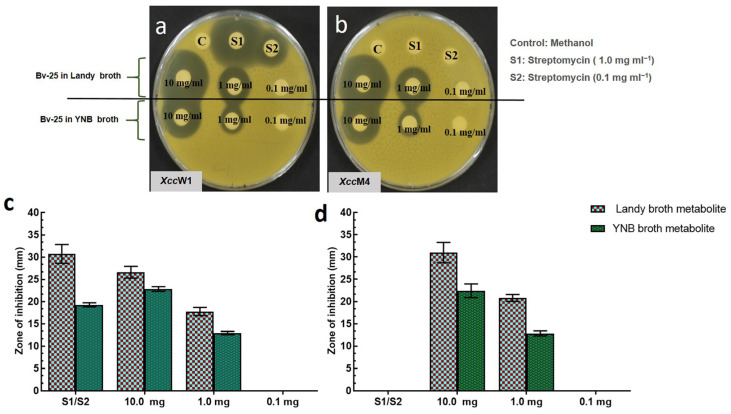
Antibacterial effect of the metabolic extract of *B. velezensis* Bv-25 against *X. citri* subsp. *citri* using the culture broth of Landy broth and YNB. (**a**) bactericidal activity was conducted against the wild-type *X. citri* subsp. *citri* (*Xcc*W1); (**b**) bactericidal activity was conducted against the streptomycin resistant *X. citri* subsp. *citri* (*Xcc*M4); C: methanol; S1: streptomycin (1.0 mg mL^−1^); S2: streptomycin (0.1 mg mL^−1^). (**c**) measurement of inhibition zones (mm) of ethyl acetate extracts of Bv-25 against *Xcc*W1; (**d**) measurement of inhibition zones (mm) of ethyl acetate extracts of Bv-25 against *Xcc*M4.

In the disk diffusion assays, ethyl acetate extracts of Bv-25 were screened for their antibacterial potentiality against the wild-type *Xcc*W1 and streptomycin resistant *Xcc*M4 strains (Figure 1). The metabolite extracted from Bv-25 using Landy broth showed higher antibacterial activity against both *Xanthomonas* strains than the one extracted using YNB. At a concentration of 10.0 mg mL^−1^ (extracted from Landy broth), the zones of inhibition for Bv-25 metabolites against *Xcc*W1 and *Xcc*M4 were 26.59 ± 1.29 mm and 30.97 ± 2.3 mm, respectively. At a concentration of 1.0 mg mL^−1^, these metabolites inhibited *Xcc*W1 and *Xcc*M4 with zones of inhibition of 17.75 ± 0.91 mm and 20.81 ± 0.78 mm, respectively. On the other hand, the metabolites that was isolated from Bv-25 using YNB broth displayed a substantially reduced zone of inhibition in comparison to the Landy broth extracted metabolites. The zones of inhibition against *Xcc*W1 and *Xcc*M4 at a dose of 10 mg mL^−1^ were 22.83 ± 0.53 and 22.43 ± 1.53 mm, respectively. For the same metabolites at a dose of 1 mg mL^−1^, the zones of inhibition against *Xcc*W1 and *Xcc*M4 were 12.91 ± 0.37 and 12.84 ± 0.55 mm, respectively. The metabolites extracted from Ba-2 did not exhibit any antibacterial effect against *Xanthomonas* pathogens.

Streptomycin concentration S1 and S2 had antagonistic effects on the wild-type *Xcc*W1, with the zone of inhibition 30.68 ± 2.12 mm and 19.26 ± 0.46 mm, respectively. However, S1 and S2 did not exhibit any antibacterial effect on the streptomycin-resistant *Xcc*M4 strain (Figure 1). 

### 3.2. Determination of MIC and MBC of the Ethyl Acetate Extracts of Bv-25 and Ba-2 Incubated in Landy Broth and YNB

MIC and MBC of crude ethyl acetate extract of strain Bv-25 were determined by testing against two *Xanthomonas* pathogens, *Xcc*W1 and *Xcc*M4, using the broth micro-dilution method. For Bv-25 extract using Landy broth culture, the MIC and MBC were 31.25 µg mL^−1^ and 62.5 µg mL^−1^, respectively against two *Xcc* strains. On the other hand, MIC and MBC of Bv-25 metabolites using YNB culture was 62.5 µg mL^−1^ and 125.0 µg mL^−1^, respectively. MIC and MBC of streptomycin (control antibiotic) against *Xcc*W1 ranged from 1.25–2.5 µg mL^−1^, but MIC and MBC of streptomycin against the streptomycin-resistant *Xcc*M4 strains were significantly high and ranged from >500–1000 µg mL^−1^ (Table 1). Metabolite extracted from Ba-2 had no effect on any *Xanthomonas* strains. 

### 3.3. Detection of Antimicrobial Compounds by HPLC-MS Analysis

HPLC-MS analysis was performed to identify the differentially produced molecules, based on the molecular weight from the MS database (Mass Spectrometry Data Center, NIST, Gaithersburg, MD, USA). These compounds mostly belonged to the class of LPs (iturin, fengycin, bacillomycin-D, and surfactin) and PKs (oxydifficidin, macrolactin, and bacillaene) (Table 2). HPLC-MS analyses indicated the presence of several active substances with protonated molecular ions at *m*/*z* 1036.6, corresponding to surfactin isoforms. The molecular mass of bacillomycin-D in the range *m*/*z* 1008–1036 matched with the molecular database. Notably, these results corresponded to the MS data obtained from the previous reports [29]. 

### 3.4. Differential Gene Expression Analysis of Bv-25 Grown in Landy and YNB Media

**Figure 2:**
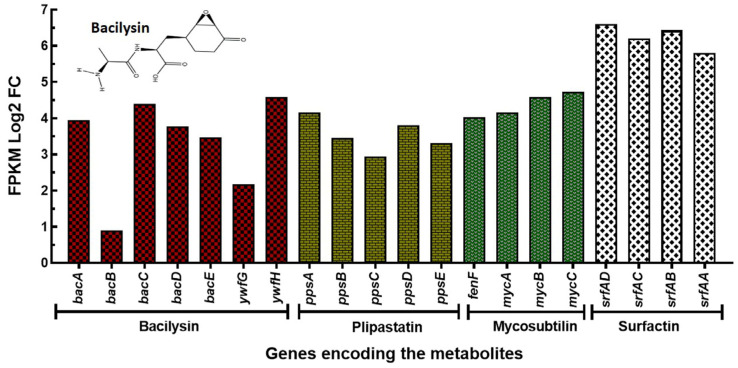
mRNA expression analysis of the biosynthetic gene clusters encoding the antimicrobial compounds of Bv-25. The relative FPKM expression level was exhibited as log2 fold change (log2 FC). FPKM (fragments per kilobase of exon model per million mapped reads).

To reveal the transcriptomic correlates of overproduction, we performed transcriptome profiling via RNA-sequencing of Bv-25 in Landy broth and YNB with an incubation period of 3 days at 28 °C. Although most genes were medially expressed in both Landy and YNB throughout the cultivation period, genes were expressed at a higher level in Landy broth. A total of 3930 genes were expressed during the mRNA expression analysis. Based on the expression data, genes encoding bacilysin (*bac*), plipastatin (*pps*) or fengycin (*fen*), surfactin (*srf*), and mycosubtilin (*myc*) were increased based on fragments per kilobase of exon model per million mapped reads (FPKM) value (Figure 2, Table 3). Besides a substantial number of genes or gene clusters involved in rhizosphere adaptation in plant beneficial traits through the formation of biofilm or by assimilating nitrogen, iron (through secretion of siderophore bacillibactin), potassium, manganese (Mn^2+^), and magnesium (Mg^2+^). Numerous genes related to plant growth promotion and induction of systemic resistance in plants were found to be expressed during mRNA expression study and are summarized in Table 4 and Table 5.

### 3.5. Antibacterial Activity Assay of Pure Surfactin and Fengycin against Xcc

By analyzing the metabolome of Bv-25, several antimicrobial substances, including surfactin, fengycin or plipastatin, and macrolactin, were detected. Therefore, we checked the antibacterial activity of fengycin, surfactin, and macrolactin; however, we were unable to detect any antibacterial impact of these compounds against the *Xanthomonas* spp. (*Xcc*W1 and *Xcc*M4).

## 4. Discussion

Biological control of diseases by *Bacillus* spp. have been extensively used to combat plant pathogenic microbes due to their specific characteristics of heat and desiccation resistance that help in the formulation of stable and dry powder with longer shelf lives. Furthermore, *Bacillus* spp. are less toxic to the environment, are capable of producing antibiotics, spores, and biofilm, and induce systemic resistance in plants [38]. Metabolite produced by a particular microorganism is crucial in determining its efficacy to be a potential biocontrol agent to combat plant diseases. In this study, we showed that the ethyl acetate extracts of Bv-25 exhibited strong antibacterial activity against both wild type and streptomycin resistant *Xcc*. Furthermore, metabolite extracted from Landy broth incubation exerted stronger antibacterial activity than that extracted from YNB incubation. 

HPLC-MS data obtained during this study showed that the antagonistic isolates were able to produce a variety of LPs (i.e., surfactins, bacilliomycin-D, and fengycin) and PKs type molecules (i.e., difficidin, bacillaene, and macrolactin). The result of this study also agree with previous investigations, which reported the presence of several non-ribosomally synthesized LPs- and PKs-type molecules in *B. velezensis* that showed antibacterial activity against phytopathogenic microbes [39]. These compounds have been investigated for their antagonistic effects against a wide variety of phytopathogenic microorganisms including *Xanthomonas oryzae* pv. *oryzae* and *X. oryzae* pv. *oryzicola* (bacterial blight and bacterial leaf streak disease of rice, respectively) [29], *Rhizoctonia solani* (bottom rot of lettuce) [40], and *X. campestris* pv. *cucurbitae* (bacterial leaf spot of cucurbits) [41]. Co-culture of *B. velezensis* FZB42 and pathogenic strain of *X. campestris* pv. *campestris* had antagonistic effect on the pathogen that causes black rot disease on cruciferous vegetables [42].

Transcriptome analysis of Bv-25 revealed the differentially increased expression of several gene clusters encoding bacilysin (*bac*), plipastatin (*pps*) or fengycin (*fen*), surfactin (*srf*), and mycosubtilin (*myc*) in the Landy broth incubation in comparison to YNB incubation. Bacilysin produced by *B. velezensis* is a dipeptide antibiotic, which relies on peptide transporters for uptake into the target cells and causes changes in the cell wall structure and efflux of intracellular components of *X. oryzae* pv. *oryzae* and *X. oryzae* pv. *oryzicola* after 12 h of exposure [29]. This dipeptide antibiotic also reported to show antifungal activity against *Candida albicans*. Upon the transportation of bacilysin into the *C. albicans* cells, intracellular proteinase hydrolyzes bacilysin into anticapsin and L-alanine. Anticapsin interacts with glucosamne-6-phosphate synthase of *C. albicans*, which is essential for cell wall formation [13,43]. 

Plipastatin or fengycin, a well-studied lipopeptide at a genetic level, is known to develop antifungal activity against filamentous fungi. Plipastatin A, secreted by *B. amyloliquefaciens* S76-3, displayed strong fungicidal activity against *Fusarium graminearum* by completely killing the conidial spores at the MIC range of 100 µg mL^−1^. Microscopy analyses revealed severe morphological changes in conidia and substantial distortions in *F. graminearum* hyphae and caused vacuolation [44]. Mycosubtilin is a potent LP-type antifungal peptide compound, mainly detected in *B. subtilis*. This compound is characterized by a β-amino fatty acid moiety linked to the circular heptapeptide [45]. Surfactins produced by *B. subtilis* 9407, which includes C13- to C16-surfactin A, were the primary antibacterial compound against *Acidovorax citrulli* (syn. *Acidovorax avenae* subsp. *citrulli*), which cause bacterial fruit blotch of cucurbit crops. A *srfAB* deletion mutant strain of *B. subtilis* 9407 ∆*srfAB*, was unable to synthesize surfactin as well as showed reduced ability to form biofilms, swarming motility and root colonization during in vivo experiments [46]. 

Based on the metabolome and transcriptome analysis, we prepared the commercially available pure fengycin and surfactin and an isolated compound macrolactin from Bv-25. We found the antagonistic effect against *Xcc* to be non-existent, therefore, there are only few *B. velezensis*-producing compounds left, including bacilysin, difficidin, bacillaene, bacillomycin D, and iturin. We assumed that the most promising candidate could be bacilysin because the activity of ethyl acetate decreased after only one week, indicating the most active compound is unstable due to presence of epoxy group on bacilysin. The unstable nature of this compound can be the most difficult challenge for agricultural application. Collectively, this study indicates that *B. velezensis* could be a promising candidate to control citrus canker caused by *X. citri* subsp. *citri* and may provide an effective strategy to combat plant pathogens.

## 5. Conclusions

In this study, we investigated the antibacterial compounds synthesized by *B. velezensis* Bv-25 against the phytopathogenic *Xcc*. Our combined investigation by HPLC-MS and differential gene expression analysis identified several LPs and PKs type antimicrobial compounds, as well as a dipeptide antibiotic, bacilysin. We assumed that bacilysin produced by *B. velezensis* could be the controlling agent against *Xcc*. Though bacilysin has been identified as a strong antimicrobial agent, the epoxy group in the compound makes it unstable. In addition to the investigation of freshly prepared bacilysin for the antagonistic effect against *Xcc*, future work will focus on the stabilization of bacilysin activity by modifying the molecular structure to interfere with the spontaneous degradation of bacilysin.

## Figures and Tables

**Table 1:** Determination of the MIC and MBC of the metabolic extract of *B. velezensis* Bv-25 against wild type *Xcc*W1 and streptomycin resistant *Xcc*M4 using the culture broth of Landy and YNB.

Xcc Strains	Extract of Bv-25 Using Landy Broth		Extract of Bv-25 Using YNB		Streptomycin	
	MIC (µg mL^−1^)	MBC (µg mL^−1^)	MIC (µg mL^−1^)	MBC (µg mL^−1^)	MIC (µg mL^−1^)	MBC (µg mL^−1^)
XccW1	31.25	62.5	62.5	125.0	1.25	2.5
XccM4	31.25	62.5	62.5	125.0	>500	>1000

**Table 2:** Cyclic lipopeptide and polyketide molecules produced by the *B. velezensis* Bv-25 strain as detected by HPLC-MS analysis. The values of Ba-2 and Bv-25 were the means of the data from three independent replications.

Compound Name	M/Z	RT (min)	Ba-2	Bv-25	Fold Change (Bv-25/Ba-2)	*p*-Value
Oxydifficidin	559.0	43.3	216,380.3	30,417,069.1	140.6	0.0027
Bacillaene	582.2	31.3	291,622.7	2,774,552.5	9.5	0.0001
Macrolactin	524.2	38.6	308,031.1	2,211,408.9	7.2	0.0046
Surfactin	1036.6	27.0	93,166.2	1,420,585.5	15.2	0.0000
Fengycin A	1475.9	30.5	128,474.2	1,092,443.5	8.5	0.0006
Fengycin B	1489.3	31.3	158,266.9	983,475.3	6.2	0.0005
Bacillomycin D	1118.6	40.5	92,804.4	19,132,037.6	206.2	0.0031
Iturin	1083.7	29.3	272,286.6	2,513,178.3	9.2	0.0036

**Table 3:** Genes associated with the antibacterial compounds of *B. velezensis* Bv-25 during mRNA expression analysis.

Active Compound	Gene	UniProt ID	Product
Bacilysin	*bacA*	Q8KWT6	Prephenate decarboxylase
	*bacB*	Q8KWT5	3-[(4R)-4-hydroxycyclohexa-1,5-dien-1-yl]-2-oxopropanoate isomerase
	*bacC*	Q8KWT4	Dihydroanticapsin dehydrogenase
	*bacD*	Q8KWT3	L-alanine-anticapsin ligase
	*bacE*	Q8KWT2	Putative bacilysin exporter
	*ywfG*	P39643	Probable aspartate aminotransferase
	*ywfH*	P39644	Bacilysin biosynthesis oxidoreductase
Plipastatin or Fengycin	*ppsA*	P39845	Plipastatin synthase subunit A
	*ppsB*	P39846	Plipastatin synthase subunit B
	*ppsC*	P39847	Plipastatin synthase subunit C
	*ppsD*	P94459	Plipastatin synthase subunit D
	*ppsE*	O31827	Plipastatin synthase subunit E
Mycosubtilin	*fenF*	Q9R9J2	Malonyl CoA-acyl carrier protein transacylase
	*mycA*	Q9R9J1	Mycosubtilin synthase subunit A
	*mycB*	Q9R9J0	Mycosubtilin synthase subunit B
	*mycC*	Q9R9I9	Mycosubtilin synthase subunit C
Surfactin	*srfAD*	Q08788	Surfactin synthase thioesterase subunit
	*srfAC*	Q08787	Surfactin synthase subunit 3
	*srfAB*	Q04747	Surfactin synthase subunit 2
	*srfAA*	P27206	Surfactin synthase subunit 1

**Table 4:** Genes associated with the bacterium-plant interactions during mRNA expression analysis of *B. velezensis* Bv-25.

Bioactivity	Gene	Product	Log2 FC
Biofilm formation (Exopolysaccharide component)	*epsC*	Probable polysaccharide biosynthesis protein	−3.17
	*epsD*	Putative glycosyltransferase	−2.08
	*epsE*	Putative glycosyltransferase	−4.00
	*epsF*	Putative glycosyltransferase	−0.45
	*epsG*	Transmembrane protein	0.04
	*epsH*	Putative glycosyltransferase	−1.25
	*epsI*	Putative pyruvyl transferase	−0.85
	*epsJ*	Uncharacterized glycosyltransferase	0.51
	*epsK*	Uncharacterized membrane protein	0.20
	*epsL*	Uncharacterized sugar transferase	−0.88
	*epsM*	Putative acetyltransferase EpsM	−1.37
	*epsN*	Putative pyridoxal phosphate-dependent aminotransferase	−0.55
Plant growth promotion and ISR induction (3- hydroxy-2-butanone)	*alsR*	HTH-type transcriptional regulator	2.77
	*alsS*	Acetolactate synthase	−3.58
	*alsD*	Alpha-acetolactate decarboxylase	−7.67
Plant growth promotion (Indole acetic acid)	*ysnE*	Uncharacterized N-acetyltransferase	inf
	*yhcX*	Hydrolase	−0.48
Plant growth promotion (Trehalose)	*treR*	Trehalose operon transcriptional repressor	1.91
	*treA*	Trehalose-6-phosphate hydrolase	1.32
	*treP*	PTS system trehalose-specific EIIBC component	0.72

**Table 5:** Expression of key genes of *B. velezensis* Bv-25 related to ion assimilation during mRNA expression analysis.

Bioactivity	Gene	Product	Log2 FC
Bacillibactin for iron (Fe^2+^) assimilation	*dhbA*	2,3-dihydro-2,3-dihydroxybenzoate dehydrogenase	0.80
	*dhbC*	Isochorismate synthase	−1.52
	*dhbE*	2,3-dihydroxybenzoate-AMP ligase	−2.45
	*dhbB*	Isochorismatase	−3.08
	*dhbF*	Dimodular nonribosomal peptide synthase	−1.32
Manganese (Mn^2+^) assimilation	*mntH*	Divalent metal cation transporter	3.71
	*mntP*	Putative manganese efflux pump	0.69
	*mntR*	Transcriptional regulator	−0.75
Magnesium (Mg^2+^) assimilation	*mgtE*	Magnesium transporter	2.19
	*corA*	Magnesium transport protein	−2.66
Potassium (K) assimilation	*ktrD*	Ktr system potassium uptake protein D	1.06
	*ktrC*	Ktr system potassium uptake protein C	−2.75
Nitrogen (N) assimilation	*moaA*	Cyclic pyranopterin monophosphate synthase	−1.03
	*moaB*	Molybdenum cofactor biosynthesis protein B	1.59
	*moaC*	Cyclic pyranopterin monophosphate synthase	0.79
	*moaD*	Molybdopterin synthase sulfur carrier subunit	0.00
	*nasB*	Assimilatory nitrate reductase electron transfer subunit	1.34
	*nasD*	Nitrite reductase [NAD(P)H]	2.82
	*nasF*	Uroporphyrinogen-III C-methyltransferase	0.41
	*nrgB*	Nitrogen regulatory PII-like protein	−3.03
	*narK*	Nitrite extrusion protein	3.04

## Data Availability

Not applicable.
